# Supplementary material for: Development of chemokine network inhibitors using combinatorial saturation mutagenesis
Source: Commun Biol. 2025 Apr 3;8:549. doi: 10.1038/s42003-025-07778-6 (PMC11969024; doi:10.1038/s42003-025-07778-6)
Supplement: Supplementary file 4 — Supplementary Data 2 [file 42003_2025_7778_MOESM4_ESM.zip › SupplementaryData3/scripts/xattune_dr.tar!/./script/analysis/JK/JK_E67_CCL3_THP1_plot.pdf]

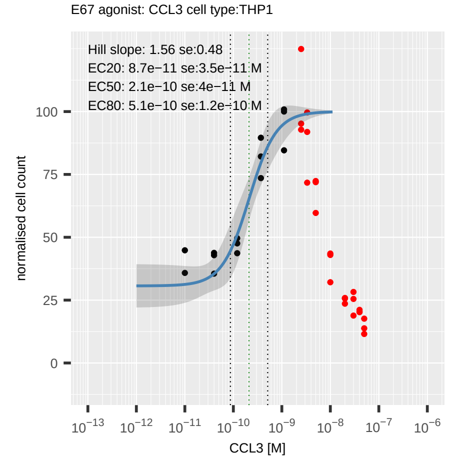
E67 agonist: CCL3 cell type:THP1
Hill slope: 1.56 se:0.48
EC20: 8.7e−11 se:3.5e−11 M
EC50: 2.1e−10 se:4e−11 M
EC80: 5.1e−10 se:1.2e−10 M
normalised cell count
0
25
50
75
100
10^{-13}
10^{-12}
10^{-11}
10^{-10}
10^{-9}
10^{-8}
10^{-7}
10^{-6}
CCL3 [M]
